# Improved Rectification and Osmotic Power in Polyelectrolyte-Filled Mesopores

**DOI:** 10.3390/mi11100949

**Published:** 2020-10-21

**Authors:** Ding-Cheng Zheng, Li-Hsien Yeh

**Affiliations:** Department of Chemical Engineering, National Taiwan University of Science and Technology, Taipei 10607, Taiwan; s088021628@gmail.com

**Keywords:** mesopore, micro/nanofluidics, ion current rectification, salinity gradient power

## Abstract

Ample studies have shown the use of nanofluidics in the ionic diode and osmotic power generation, but similar ionic devices performed with large-sized mesopores are still poorly understood. In this study, we model and realize the mesoscale ionic diode and osmotic power generator, composed of an asymmetric cone-shaped mesopore with its narrow opening filled with a polyelectrolyte (PE) layer with high space charges. We show that, only when the space charge density of a PE layer is sufficiently large (>$1\times 10^{6}{\;C/m}^{3}$), the considered mesopore system is able to create an asymmetric ionic distributions in the pore and then rectify ionic current. As a result, the output osmotic power performance can be improved when the filled PE carries sufficiently high space charges. For example, the considered PE-filled mesopore system can show an amplification of the osmotic power of up to 35.1-fold, compared to the bare solid-state mesopore. The findings provide necessary information for the development of large-sized ionic diode and osmotic power harvesting device.

## 1. Introduction

Nanofluidics, such as nanopores and nanochannels [1,2,3], has been extensively studied, because unique ion transport properties are expected to emerge from the overlapping effect of electric double layers (EDLs) in confined nanospaces, making it with diverse applications from energy, to environment, to biosensors [4,5,6,7,8,9,10,11,12,13,14,15,16,17]. For example, the diode-like ion current rectification (ICR) behavior, which assures ions preferentially transporting in one direction and hence amplifies ionic current, can be observed when the symmetry of ionic concentration profiles along the axis of a nanopore is broken [18,19]. Considerable experimental [20,21,22] and theoretical [23,24,25,26,27,28,29,30,31,32] efforts have been made on ICR in nanofluidics and all these studies concluded that the ICR property only can emerge in case the pore size is comparable to the EDL thickness.

One of the popular nanofluidic applications is the osmotic power generation [33,34,35], in which the energy stored in a salinity gradient can be converted into electricity with a high conversion efficiency. Recent advances in material science and nanotechnology improved the output performance of the nanofluidic osmotic power generators across over the commercial benchmark (5 W/m^2^) [36,37,38]. Even though such a big progress has been made [36,37,38] and there have been several reports showing that the nanofluidic osmotic power performance can be further improved by the incorporation of ICR effect [35,39,40,41], nanopores and nanochannels have still been suffering from high mass transport resistance from such tiny pore sizes, thus limiting the breakthrough of osmotic power into the practical industry application.

Until recently, our group demonstrated that the ICR property can appear in an asymmetric mesopore with a pore size of ~400 nm [42,43]. The fact to induce the mesoscale ICR results from the high surface charged pore walls, which in turn create the broken symmetry and the asymmetric ionic distributions along the axis of a pore. The effect of ICR was further used to create a mesoscale osmotic power generator by filling a poly-L-lysine layer with high space charges into the interior of a ~400 nm-in-tip-diameter mesopore [41]. It was experimentally shown that a significant ICR effect can be found in neutral and high saline solutions, capable of achieving an osmotic power of ~120 pW under a 500-fold salinity gradient. This opens a pathway towards high-performance osmotic power harvesting, and thus, it is highly necessary to simulate the ion transport and osmotic power conversion in the relevant mesopore system, which is able to improve the understanding of underlying mechanisms behind the mesoscale transport.

Here, we theoretically investigate the mesoscale ionic diode and osmotic power generator by considering a cone-shaped mesopore whose narrow opening is filled with a positively charged polyelectrolyte (PE) layer. Compared with all the earlier theoretical works focusing primarily on the nanofluidic powers [44,45,46,47], this is the first report studying the osmotic power generator at the mesoscale. The effect of the most key parameter to the system, the space charge density of a PE layer ($\rho_{PE}$), on the rectification ability and osmotic power conversion is systematically discussed. It is found that the performance of the mesoscale osmotic power harvesting system considered can be significantly improved when the filled PE layer carries sufficiently high space charges. The finding provides significant information for exploration of novel relevant mesoscale devices in the future.

## 2. Theoretical Model

**Figure 1:**
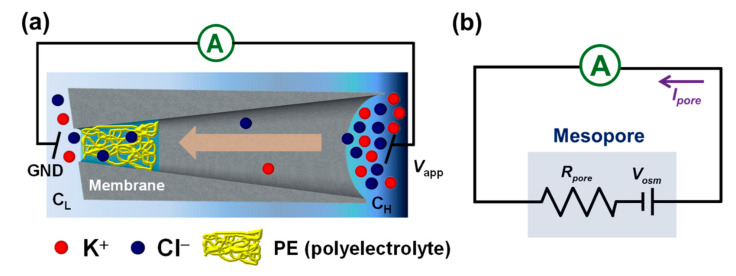
(**a**) Schematic depiction and (**b**) the corresponding equivalent electric circuit of the mesoscale osmotic energy harvesting system under consideration. The electrode in the low concentration reservoir is grounded (GND), while is applied at a voltage $V_{app}$ in the high concentration reservoir. $R_{pore}$ is the pore resistance, $V_{osm}$ is the osmotic voltage caused by a concentration gradient across a mesopore, and $I_{pore}$ is the generated ionic current through the mesopore.

Figure 1a depicts the schematic view of the mesoscale osmotic power harvesting system under consideration, where we consider a cone-shaped mesopore with 200 nm in tip radius and a half cone angle of 4° [41], and its narrow opening is filled with a space-charged PE layer of 200 nm in thickness. It is known that the mesopore can be fabricated in a solid-state polymer (e.g., polyethylene terephthalate, PET) membrane and carries negative surface charges when it is in contact with aqueous solution because of the existence of carboxyl functional groups on the pore wall; therefore, we assume that the PE layer carries a positive space charge density of uniformly distributed $\rho_{PE}$. We let the mesopore connect two large, identical reservoirs of length $L_{r}$ and radius $R_{r}$, and assume the reservoir outside the narrow opening is filled with a KCl solution of a lower bulk salt concentration $C_{L}$ and grounded, while the other reservoir outside the base opening is filled with a KCl solution of a higher bulk salt concentration $C_{H}$ and applied at a voltage bias $V_{app}$. The equivalent electric circuit of the mesopore system under consideration can be found in Figure 1b. In case the space close to the narrow opening of a mesopore is occupied by a positively charged PE, the number of anions passing through the pore, driven by a salinity gradient, will be higher than that of cations, inducing an osmotic current ($I_{osm}$) and an osmotic voltage ($V_{osm}$).

Suppose that the liquid phase is an incompressible Newtonian fluid. The ion transport in the mesopore system considered can be described by the coupled Poisson–Nernst–Planck and Stokes–Brinkman equations, taking account of the space charge density stemming from the PE layer, which have been validated for the nanopore systems [48,49,50,51]:

(1) $$-\nabla^{2}\phi =\;\frac{\rho_{e}+h\rho_{PE}}{\varepsilon_{f}},$$

(2) $$\nabla \cdot J_{i}=\nabla \cdot \left[vC_{i}-D_{i}\nabla C_{i}-\left(\frac{Fz_{i}C_{i}D_{i}}{RT}\right)\nabla \phi \right]=0,\;i=1,\;2\;$$

(3) $$-\nabla p+\mu \nabla^{2}v-\rho_{e}\nabla \phi -\frac{h\mu v}{{\left(\lambda_{PE}\right)}^{2}}=0,$$

(4) ∇⋅v=0. 

In the above, $\rho_{e}=\sum_{i=1}^{2}Fz_{i}C_{i}$ is the space charge density of mobile ions; ϕ is the electric potential; $\varepsilon_{f}$, R, F, and T are the fluid permittivity, gas constant, Faraday constant, and absolute fluid temperature, respectively; $C_{i}$, $D_{i}$, $J_{i}$, and $z_{i}$ are the concentration, diffusivity, flux, and valence of the *i*^th^ ionic species (i=1 for cations and i=2 for anions), respectively; **v** and μ are the velocity and viscosity of fluid, respectively; *p* is the hydrodynamic pressure; $\lambda_{PE}={\left(\mu /\gamma_{PE}\right)}^{1/2}$ is the softness degree of the PE layer; $\gamma_{PE}$ is the hydrodynamic frictional coefficient of the PE layer; *h* is the space region function (h=1 for the region inside the PE layer; h=0 for the region outside the PE layer). Considering Equations (3) and (4) into the model indicates that the effect of the electrodiffusioosmotic flow [52,53], which plays a role at the mesoscale, has been considered.

The boundary conditions for Equations (1)–(4) are as follows. (i) The ionic concentrations at the ends of the two reservoirs reach their bulk values; that is, $C_{i}=C_{H}$ (right reservoir) and $C_{i}=C_{L}$ (left reservoir). The corresponding electric potentials are specified as $\phi =V_{app}$ (right reservoir) and ϕ=0 (left reservoir), and there is no pressure gradient across the reservoirs. (ii) The rigid membrane walls are uncharged, ion-impenetrable, and nonslip, yielding n⋅∇ϕ=0, $n\cdot J_{i}=0$, and v=0, respectively. Here, **n** is the unit outer normal vector. (iii) An insulation boundary condition for the electric potential (n⋅∇ϕ=0), zero normal ionic fluxes ($n\cdot J_{i}=0$), and slip boundary condition for the flow field are specified at the both side boundaries of the two reservoirs. (iv) The electric potential, electric field, ionic concentrations, and flow field are all continuous on the liquid–PE layer interfaces [48].

In the absence of a voltage bias and/or a concentration gradient through a mesopore, the resulting ionic current can be evaluated by

(5) $$I_{pore}=\int_{S}(\sum_{i=1}^{2}Fz_{i}J_{i})\cdot ndS,\;$$

where *S* denotes the either end of the reservoirs. Then the transference number of anions ($t_{an}$) under a concentration gradient is calculated as [44]

(6) $$t_{an}=\frac{\left|I_{2}\right|}{\left|I_{1}\right|+\left|I_{2}\right|},\;$$

where $I_{1}$ and $I_{2}$ are the osmotic current contributed from cations and anions, respectively. In general, the more significant deviation of $t_{an}$ from 0.5 to 1, the more significant the anion selectivity.

**Figure 2:**
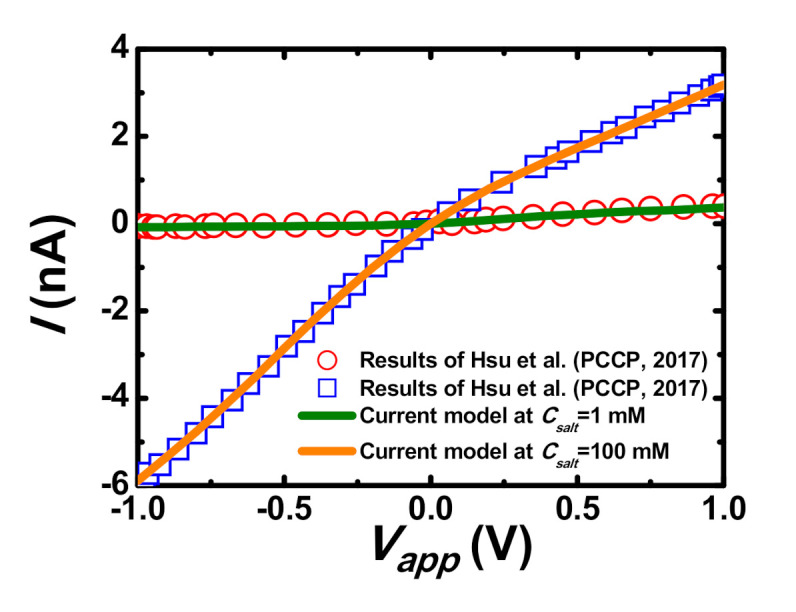
Code validation by comparing the results from the current model (solid curves) with the previous results of Hsu et al. [57], who modeled the current-voltage curves of a polyelectrolyte (PE)-modified nanopore at the two levels of KCl concentrations.

Coupled and highly nonlinear Equations (1)–(4) along with the above-mentioned boundary conditions are solved numerically by the commercial finite element software, COMSOL Multiphysics (version 4.3a, COMSOL, Stockholm, Sweden). Finer mesh is generated in the PE plug layer region and the mesh-independent test is performed to ensure all results are sufficiently accurate and reliable. Typically, the number of total meshes used is around 228,000. Although the applicability of the model adopted has been validated in our previous studies of ion transport in nanopores with PE coatings [48,49,50,54,55,56], its applicability is also further confirmed by fitting it to the current–voltage curves of a PE-modified conical nanopore at the two levels of KCl concentrations [57]. As shown in Figure 2, the predicted results from our current model agree well with the results of Hsu et al. [57].

## 3. Results and Discussion

In the subsequent discussions, we fix the following physical parameters at T=298 K: $D_{1}(K^{+})=1.96\times 10^{-9}{\;m}^{2}/s$, $D_{2}({\mathrm{Cl}}^{-})=2.03\times 10^{-9}{\;m}^{2}/s$, $\varepsilon_{f}=78.5\times 8.854\times 10^{-12}\;F/m$, and $\mu =10^{-3}\;\mathrm{Pa}\cdot s$ [58]. The geometry of the bare solid-state cone-shaped mesopore adopted is $R_{t}=200\;\mathrm{nm}$, $R_{b}=480\;\mathrm{nm}$, and $L_{n}=4000\;\mathrm{nm}$. The size of the two reservoirs is assumed to be large enough, *i.e.*, $R_{r}=1200\;\mathrm{nm}$ and $L_{r}=1200\;\mathrm{nm}$, so that the effect of the reservoir size on the ionic current results can be neglected [59]. We then fix the softness degree of the PE layer $\lambda_{\mathrm{PE}}=1\;\mathrm{nm}$, in accordance with the typical values of synthetic PEs (*ca.*, 0.1–10 nm) [60,61,62]. This study focuses on the influence of the space charge density of a PE layer, the most crucial factor in the mesopore system considered, on the ion transport and osmotic power conversion in a PE-filled mesopore.

### 3.1. Modeling of Mesoscale Ionic Diode

**Figure 3:**
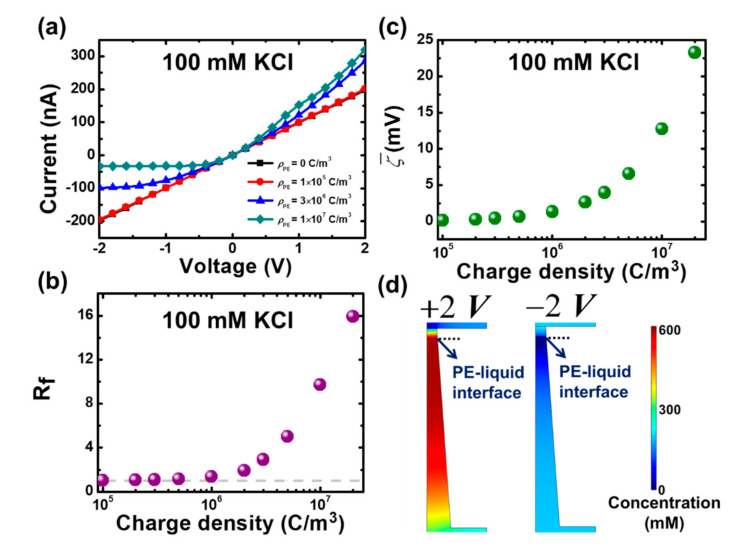
(**a**) Simulated current–voltage (I–V) curves of the considered mesopore system with various space charge densities ($\rho_{PE}$ ) at $C_{salt}=100\;\mathrm{mM}$. (b) Rectification ratio ($R_{f}$) and (**c**) the averaged zeta potential ($\bar{\zeta }$) as a function of $\rho_{PE}$ at $C_{salt}=100\;\mathrm{mM}$. The dashed curve in (b) is the rectification ratio ($R_{f}=1$) for the bare mesopore system in the absence of a PE layer. (**d**) Spatial variations of the total ion concentration in the considered mesopore system with $\rho_{PE}=1\times 10^{7}{\;C/m}^{3}$ for the two applied voltages with opposite signs at $C_{salt}=100\;\mathrm{mM}$.

We first investigate the ion transport of the considered mesopore system in the absence of a concentration gradient *(i.e.,* $C_{H}=C_{L}=C_{\mathrm{salt}}$). Figure 3a illustrates the simulated I–V curves of the considered mesopore system in the absence ($\rho_{\mathrm{PE}}=\gamma_{\mathrm{PE}}=0$) and presence of a PE plug layer at $C_{salt}=100\;\mathrm{mM}$ and the calculated rectification ratio, $R_{f}=\left|I\left(+2\;V\right)/I\left(-2\;V\right)\right|$, is depicted in Figure 3b. Considering such a high 100 mM salt concentration, corresponding to the EDL thickness of ~1 nm which is 400 times smaller than the narrow opening diameter of a mesopore considered, in the simulations is to ensure that the EDL overlap effect is no longer significant. As expected, the bare conical mesopore itself does not rectify ($R_{f}=1$), which agrees with the experimental finding [56]. This comes from the fact that the EDL in the 400 nm in tip diameter mesopore does not overlap, leading to negligible ion selectivity capable of causing asymmetric ionic concentration distributions along the axis of a pore [18]. On the contrary, if the PE layer bears a sufficiently high space charge density (e.g., >$1\times 10^{6}{\;C/m}^{3}$), the considered mesopore system starts to exhibit a diodelike ICR phenomenon (i.e., an asymmetric I–V curve where the current at a positive voltage is higher than that at a negative voltage with an opposite sign), demonstrating the realization of the mesoscale ionic diode.

The rectification of the considered mesopore system can be explained by the introduction of a high space charged PE plug layer into the asymmetric confined space, which induces a high averaged zeta potential (i.e., the electric potential at the PE/mesopore interface) (Figure 3c) and, therefore, an asymmetric distributions of ionic concentrations in the mesopore due to the ion concentration polarization (ICP) effect [41]. As shown in Figure 3d, the total ion concentration in the pore interior is accumulated at a voltage bias of +2 V but depleted at −2 V, demonstrating the asymmetric conduction state observed in Figure 3a. Our modeling presented in Figure 3 also reveals the importance of the PE plug layer. If the filling of the PE layer bears an insufficiently high charge density (e.g., <$1\times 10^{6}{\;C/m}^{3}$), the considered mesoscale pore system is still unable to exhibit an apparent ICR effect. Figure 3b also depicts that the ionic rectification ability of the considered mesopore system increases with the increase in the PE’s space charge density, suggesting the tunable performance of the mesoscale ionic diode proposed.

### 3.2. Modeling of Mesoscale Osmotic Power Conversion

**Figure 4:**
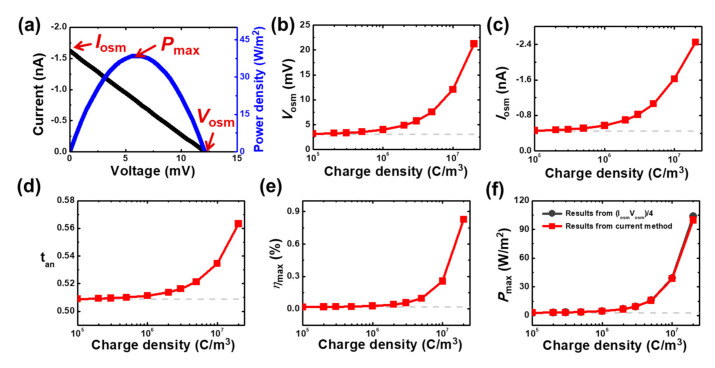
(**a**) Simulated current–voltage (I–V) and power density–voltage (P–V) curves of the considered mesopore system with a space charge density of $\rho_{PE}=1\times 10^{7}{\;C/m}^{3}$ in a 1000 mM/1 mM salinity gradient. The osmotic voltage ($V_{osm}$) and current ($I_{osm}$) can be obtained from the intercepts on the current and voltage axes, respectively. $P_{\max}$ is the maximum osmotic power density generated. Simulated (**b**) osmotic voltage, (**c**) osmotic current, (**d**) transference number of anions ($t_{an}$), (**e**) maximum energy conversion efficiency ($\eta_{\max}$), and (**f**) maximum osmotic power density ($P_{\max}$) of the considered mesopore system as a function of the PE’s space charge density in 1000 mM/1 mM salinity gradient. The dashed curves in (b)–(f) are the results from the bare solid-state mesopore system. In (f), the results of $P_{\max}$ from the conventional method, $\left(I_{osm}\times V_{osm}\right)/4$, are also presented for comparison. The lower salt concentration is fixed at 1 mM.

Since the mesoscale ionic diode considered is successfully modeled and realized (Figure 3), its application in the osmotic power conversion is then studied. According to the simulation setup and the equivalent electric circuit shown in Figure 1, the voltage bias ($V_{app}$) applied through the considered mesopore system can be described by

(7) $$V_{app}=V_{osm}-I_{pore}R_{pore},\;$$

where $I_{pore}$ is the ionic current through the mesopore and $R_{pore}$ is the pore resistance. In the absence of a voltage bias ($V_{app}=0$), the ionic current through a pore is contributed from the osmotic voltage, which is induced purely from a concentration gradient. In this case, the osmotic current is estimated as $I_{osm}=I_{pore}$. On the other hand, the osmotic voltage can be obtained by letting $I_{pore}=0$ [44]. Figure 4a illustrates the simulated current–voltage and the power density–voltage curves of the considered mesopore system in 1000 mM/1 mM salinity gradient where the power density is calculated as $P=\left|V_{app}\times I_{pore}\right|/\pi R_{t}^{2}$. The $I_{osm}$ and $V_{osm}$ can be read from the intercepts on the voltage and current axes, respectively. As can be seen in Figure 4a, the simulated current–voltage curve is slightly nonlinear, implying that it does not fully obey Ohm’s law. This can be attributed to the significant ICP effect arising from the high space charge density of a PE layer [48]. Hence, we calculate the maximum osmotic power density ($P_{\max}$) as the highest value of the P–V curve in the present manuscript, instead of using the simple formula that the previous studies adopted [44,63,64],

(8) $$P_{\max}=\frac{\left|V_{osm}\times I_{osm}\right|}{4\pi R_{t}^{2}}.$$

The influence of the space charge density of a PE layer, $\rho_{PE}$, on the $V_{osm}$, $I_{osm}$, $t_{an}$, maximum conversion efficiency ($\eta_{\max}$), and $P_{\max}$ of the considered mesoscale pore system is shown in Figure 4b–f. Here, the $\eta_{\max}$ is the efficiency corresponding to the maximum osmotic power, which can be calculated by using [44]

(9) $$\eta_{\max}=\left(2t_{an}-1\right)\frac{\left(V_{osm}/2\right)}{\left(\frac{RT}{F}\right)\ln\left(\frac{\gamma_{C_{H}}C_{H}}{\gamma_{C_{L}}C_{L}}\right)},$$

where $\gamma_{C_{H}}$ and $\gamma_{C_{L}}$ are the mean activity coefficients of electrolyte solutions in the higher and lower concentration reservoirs, respectively. Note that the corresponding results of a bare solid-state mesopore without a PE layer (dashed curves in Figure 4b–f) and the result of $P_{\max}$ estimated based on Equation (8) (black solid curve with spheres in Figure 4f) are also presented for comparison. As shown in Figure 4b–f, if $\rho_{PE}$ is small (*e.g.*, <$1\times 10^{6}{\;C/m}^{3}$), the considered mesopore system outputs almost negligible osmotic power performance as small as the bare solid-state mesopore. The findings are consistent with Figure 3b, where the PE-filled pore system with a small $\rho_{PE}$ is almost unable to rectify ionic current, implying it is nearly of no anion selectivity. On the other hand, if $\rho_{PE}$ is sufficiently large (e.g., >$1\times 10^{6}{\;C/m}^{3}$), the osmotic power performance increases sharply with an increase in $\rho_{PE}$. Compared with the bare solid-state mesopore, the osmotic power density of the considered PE-filled mesopore system can be improved from about 0.65-fold to 35.1-fold for the increase of $\rho_{PE}$ from $1\times 10^{6}$ to $2\times 10^{7}{\;C/m}^{3}$, demonstrating the realization of the mesoscale osmotic power generator. It is worth noting in Figure 4f that the osmotic power density estimated by Equation (8) is slightly larger than that by the present method from the P-V curve (see Figure 4a). For example, for $\rho_{PE}=2\times 10^{7}{\;C/m}^{3}$, an overestimation of ~3.9% is observed. The overestimation can be attributed to the highly space-charged PE layer, which induces significant ICP effect and nonlinear ion transport phenomenon [48].

**Figure 5:**
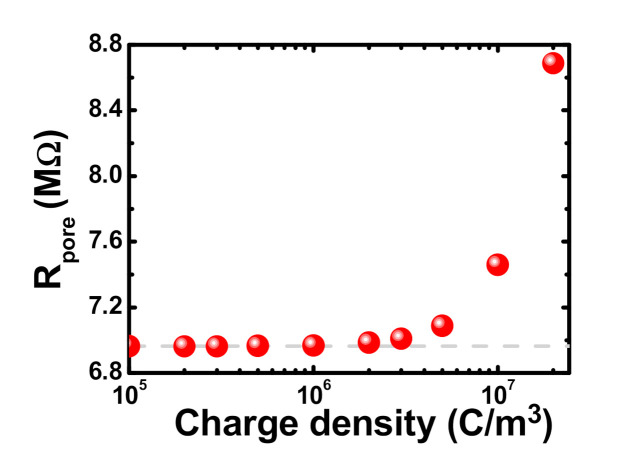
Resistance (*R_pore_*) of the considered mesopore system as a function of $\rho_{PE}$ in a 1000 mM/1 mM salinity gradient. The dashed curve represents the result from the bare solid-state mesopore system.

We also summarize the resistance of the considered mesopore system, $R_{pore}=V_{osm}/I_{osm}$, as a function of the space charge density of a PE layer, $\rho_{PE}$. As shown in Figure 5, if $\rho_{PE}$ is small, the resistance of the considered mesopore system behaves like the bare solid-state mesopore without a PE plug layer. On the contrary, the resistance of the considered mesopore system increases sharply with increasing $\rho_{PE}$ if it is sufficiently large. The charge-density-dependent $R_{pore}$ is consistent with the behaviors of $V_{osm}$, $I_{osm}$, $t_{an}$, $\eta_{\max}$, and $P_{\max}$ shown in Figure 4b–f. For sufficiently large $\rho_{PE}$, the sharp increase of $R_{pore}$ can be explained by the more significant increase of $V_{osm}$ than that of $I_{osm}$ (Figure 4b,c), due to the presence of highly space-charged PEs.

**Figure 6:**
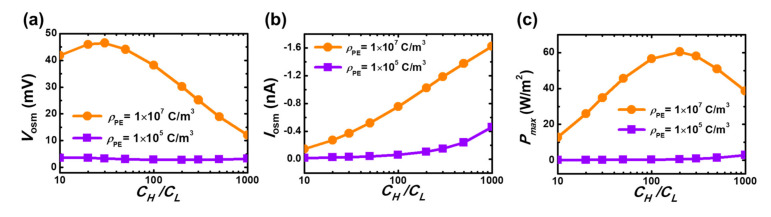
(**a**) Simulated osmotic voltage, (**b**) osmotic current, and (**c**) maximum osmotic power density as a function of the concentration gradient for the two levels of space charge densities. The lower salt concentration is fixed at 1 mM.

We then investigate the influence of a salinity gradient on the three major osmotic power output performance, $V_{osm}$, $I_{osm}$, and $P_{\max}$ in Figure 6. Here we consider two levels of space charge densities of a PE layer, $\rho_{PE}=1\times 10^{5}{\;C/m}^{3}$ and $1\times 10^{7}{\;C/m}^{3}$. Consistent with the results shown in Figure 4, the considered mesopore system with a small $\rho_{PE}$ can output negligible $V_{osm}$, $I_{osm}$, and $P_{\max}$, no matter how large the applied salinity gradient is. On the contrary for a large $\rho_{PE}$, the generated $V_{osm}$ exhibits a local maximum and $I_{osm}$ reveals a monotonic increase with the increase in the salinity gradient. The behaviors are similar to the nanoscale osmotic power harvesting systems [44,45]. In general, both the $V_{osm}$ and $I_{osm}$ increase with the increase of the concentration gradient, but the former is also governed by $t_{an}$. The larger the concentration gradient, the smaller the ion selectivity, leading to a smaller $t_{an}$. As $C_{H}/C_{L}$ increases, the osmotic voltage, if the decrease in $t_{an}$ dominates, decreases with increasing concentration gradient. Note that the considered mesoscale pore system does not have apparent ion selectivity (Figure 4d), and therefore the $V_{osm}$ decreases sharply with $C_{H}/C_{L}$ when it is sufficiently large, resulting in a local maximum dependence of $P_{\max}$ on $C_{H}/C_{L}$. The findings in Figure 4 and Figure 6 indicates two design guidelines for the development of mesoscale osmotic power generators. (i) The space charge density of a PE layer is the key for high-performance output and the mesoscale osmotic power harvesting does not function at the condition at which the PE’s space charge density is insufficiently high. (ii) The osmotic power density output can be maximized by adopting an appropriate salinity gradient, which is inconsistent with the nanofluidic osmotic power harvesting systems [44,45].

## 4. Conclusions

In summary, we model the mesoscale ionic diode and osmotic power generator, consisting of a 400 nm in tip diameter cone-shaped mesopore, and its narrow opening is filled with a PE plug layer with high space charges. We show that both the systems can be realized when the PE layer carries a sufficiently high space charge density (>$1\times 10^{6}{\;C/m}^{3}$). Otherwise, the considered PE-filled mesopore system with a small space charge density behaves like the bare solid-state mesopore. An amplification of the osmotic power ranging from 0.65-fold to 35.1-fold can be predicted for the filled PE layer carrying a space charge density raising from $1\times 10^{6}$ to $2\times 10^{7}{\;C/m}^{3}$, compared to the bare solid-state mesopore. More importantly, the considered mesopore system reveals a local maximum dependence of output osmotic power on the salinity gradient, implying that an optimization of osmotic power can be realized for the mesoscale energy harvesting system.
